# Bacterial succession and functional diversity during vermicomposting of the white grape marc *Vitis vinifera* v. Albariño

**DOI:** 10.1038/s41598-019-43907-y

**Published:** 2019-05-16

**Authors:** Allison R. Kolbe, Manuel Aira, María Gómez-Brandón, Marcos Pérez-Losada, Jorge Domínguez

**Affiliations:** 1 Computational Biology Institute, Milken Institute School of Public Health, George Washington University, Ashburn, VA 20147 USA; 2 Departamento de Ecoloxía e Bioloxía Animal, Universidade de Vigo, Vigo, E-36310 Spain; 3 CIBIO-InBIO, Centro de Investigação em Biodiversidade e Rescursos Genéticos, Universidade do Porto, Campus Agrário de Vairão, 4485-661 Vairão, Portugal; 4 Department of Epidemiology and Biostatistics, Milken Institute School of Public Health, George Washington University, Washington, DC, 20052 USA

**Keywords:** Microbiome, Applied microbiology

## Abstract

Winemaking produces millions of tons of grape marc, a byproduct of grape pressing, each year. Grape marc is made up of the skins, stalks, and seeds remaining after pressing. Raw grape marc can be hazardous to the environment due to its low pH and high polyphenol content, but previous work has shown that grape marc can be stabilized via vermicomposting to produce organic fertilizer. Here, we utilize 16S rRNA high-throughput sequencing to characterize the bacterial community composition, diversity and metabolic function during vermicomposting of the white grape marc *Vitis vinifera* v. Albariño for 91 days. Large, significant changes in the bacterial community composition of grape marc vermicompost were observed by day 7 of vermicomposting and throughout the duration of the experiment until day 91. Similarly, taxonomic and phylogenetic α-diversity increased throughout the experiment and estimates of β-diversity differed significantly between time points. Functional diversity also changed during vermicomposting, including increases in cellulose metabolism, plant hormone synthesis, and antibiotic synthesis. Thus, vermicomposting of white grape marc resulted in a rich, stable bacterial community with functional properties that may aid plant growth. These results support the use of grape marc vermicompost for sustainable agricultural practices in the wine industry.

## Introduction

Wine production is a major agricultural industry in many parts of the world. Over 70 million metric tons of grapes were produced annually worldwide^1^, of which approximately half are used to make wine^2^. Grape marc - the skins, stalks, and seeds remaining after grape pressing - makes up about 25% of the total grape weight used in the winemaking process^3^. Therefore, millions of tons of grape marc are produced per year as a result of winemaking activities. Grape marc is nutrient rich, but is also characterized by a low pH and high polyphenol content^4^, which can be both polluting for the environment and phytotoxic in agricultural applications^5^. Thus, grape marc produced during winemaking necessitates environmentally responsible disposal^6–8^.

In Galicia (northwestern Spain), 95% of grapes produced belong to the white grape variety Albariño, producing 28.6 million liters of wine annually^9^. Therefore, proper disposal of grape marc resulting from wine production with the Albariño grape is of great significance in the region. Although applying grape marc as a soil amendment would be a sustainable solution for this waste, grape marc strongly inhibits germination^9^ and is not suitable for use on agricultural lands in its raw form. White grape marc can be particularly high in phytotoxic polyphenols due to differences in the winemaking process^5^. During red winemaking, the grape marc is fermented with the grape juice, resulting in transfer of the polyphenol content from the grapes to the wine. In contrast, white wine is typically fermented with little or no contact with the grape marc^5^. Therefore, the grape marc from white winemaking retains a high polyphenol content, and proper disposal is essential to minimize deleterious effects on the environment.

Stabilization of grape marc via thermophilic composting has been explored^10,11^, but the low pH of grape marc poses challenges for large-scale composting applications of grape marc. Its low pH inhibits transitions between mesophilic and thermophilic composting phases, and requires addition of neutralizing agents before composting can begin^11^. Co-composting of grape marc with municipal wastes has been shown as a more effective alternative^5^, but requires extensive optimization based on the types of wastes and composting conditions and therefore may be difficult to enact in practice.

Vermicomposting, the mesophilic production of organic amendment via interactions between microbes and earthworms, is an attractive alternative as it may circumvent the problems encountered with composting of grape marc^5,8,12–14^. Vermicomposting has previously been shown to effectively neutralize the pH of grape marc and reduce polyphenol content and phytotoxicity^8,12–15^. Furthermore, the application of grape marc vermicompost has been shown to positively improve vineyard soil through enhanced availability of nitrogen and carbon mineralization^16^. Therefore, vermicomposting of grape marc may offer a sustainable alternative for wineries to recycle winery wastes. However, optimization of grape marc vermicomposting for commercial applications necessitates a better understanding of the underlying biological processes.

In order to assess the role of microbial communities (microbiomes) during vermicomposting of marc from an exemplar white grape (*Vitis vinifera* v. Albariño), we coupled 16S rRNA high-throughput sequencing and sophisticated metataxonomic analyses^17^ of amplicon sequence variants. Using this approach we characterized the taxonomic and phylogenetic diversity, metabolic function and explored bacterial community succession throughout the vermicomposting process for 91 days.

## Results

### Earthworm density and microbial activity during vermicomposting of white grape marc

**Figure 1:**
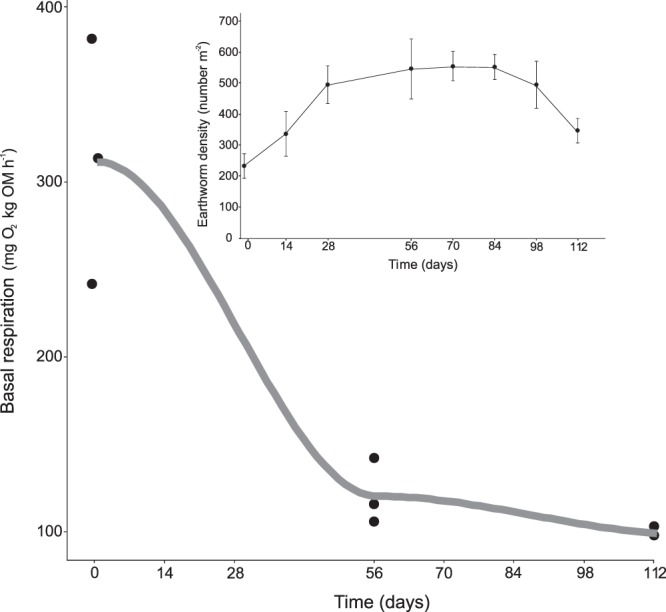
Variation in microbial respiration during vermicomposting of the white grape marc *Vitis vinifera* v. Albariño. Individual values (n = 3) are plotted for each time point, and the curve was plotted using the “loess” smoothing method in ggplot2^52^. The inset shows changes in earthworm density during the process. Earthworm density values are presented as means ± standard error (n = 5).

The total number of earthworms increased during the vermicomposting process until day 70, when the population reached its maximum density (Fig. 1 inset). Since no additional substrate was added to the vermireactor after day 0, the earthworm population increased to its maximum density and then declined until the end of the experiment. Microbial activity (measured as basal respiration) decreased during vermicomposting (Fig. 1). These results indicate that the vermicomposting process was successful and provides an adequate framework for studying the succession of bacterial communities during vermicomposting.

### Changes in bacterial community composition during vermicomposting of white grape marc

**Figure 2:**
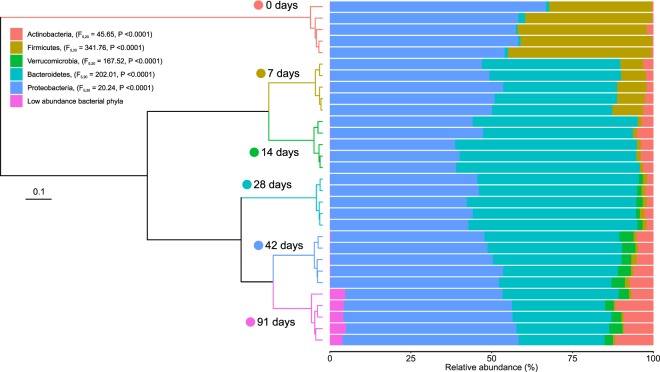
Changes in the bacterial community composition (phylum level) during vermicomposting of the white grape marc *Vitis vinifera* v. Albariño. The dendrogram represents the dissimilarity of bacterial communities at ASV levels (weighted UniFrac distances, Ward method). Bars represent the relative abundance of dominant bacterial phyla. Low abundance bacterial phyla (<1%) were grouped together.

Bacterial community composition changed significantly (p < 0.001) during vermicomposting of white grape marc (Fig. 2; Table 1). The bacterial community of fresh grape marc was dominated by the phyla Proteobacteria and Firmicutes, but large changes were observed in community composition after only 7 days of vermicomposting. Most notably, the abundance of Firmicutes, which began at nearly 40%, decreased rapidly to 8% in day 7 and remained around 1% relative abundance for the duration of the experiment (Table 1). Bacteroidetes increased significantly (p < 0.001) within the first 7 days and made up approximately 50% of sequences during the middle time points of the experiment (days 14 and 28) before decreasing slightly in the mature vermicompost. The relative abundance of sequences belonging to phyla Actinobacteria and Verrucomicrobia was small, but increased during vermicomposting.

**Table 1:** Mean relative abundances of the dominant phyla during vermicomposting of the white grape marc *Vitis vinifera* v. Albariño.

*Relative abundances (Phylum)*	Day 0	Day 7	Day 14	Day 28	Day 42	Day 91	*F*_*5,20*_	*P*(>*F*)
Actinobacteria	0.78a	2.68bc	3.92c	2.26b	5.75b	9.73d	45.65e	<0.0001
Bacteroidetes	1.09a	38.75b	52.96c	50.76c	38.66b	30.10d	202.0	<0.0001
Firmicutes	39.17a	8.38b	1.22c	1.29c	1.09c	0.61c	341.76	<0.0001
Proteobacteria	58.89a	50.14b	41.81c	44.13c	50.37b	52.00b	20.24	<0.0001
Verrucomicrobia	0.01a	0.00a	0.01a	1.48b	3.94c	3.13d	167.52	<0.0001
Low abundance	0.05	0.06	0.09	0.08	0.19	4.43	—	—

Phyla with abundance <1% were grouped together. Results from mixed-effects models analyses are shown. Significance was determined using ANOVA. For each phylum, we report the relevant *F* statistic and degrees of freedom (*F*_*5,20*_) and significance (*P(*>*F))*. Letters indicate significant differences between time points (Tukey HSD test). Degrees of freedom were constant across all tests.

Over 50% of the sequences in the initial grape marc substrate belonged to the phylum Proteobacteria. Although Proteobacteria continued to make up a significant proportion of the bacterial communities during vermicomposting, there was a shift in the composition of Proteobacteria broken into the class level (Supplementary Fig. 2). The majority of Proteobacteria sequences in the fresh grape marc belonged to the class Alphaproteobacteria (95%); however, Gammaproteobacteria made up a large portion of Proteobacteria sequences during vermicomposting (47–75%). In the case of the other four most abundant phyla during vermicomposting, each was dominated by a single class (Supplementary Fig. 2).

### Changes in α- and β-diversity during vermicomposting of white grape marc

**Figure 3:**
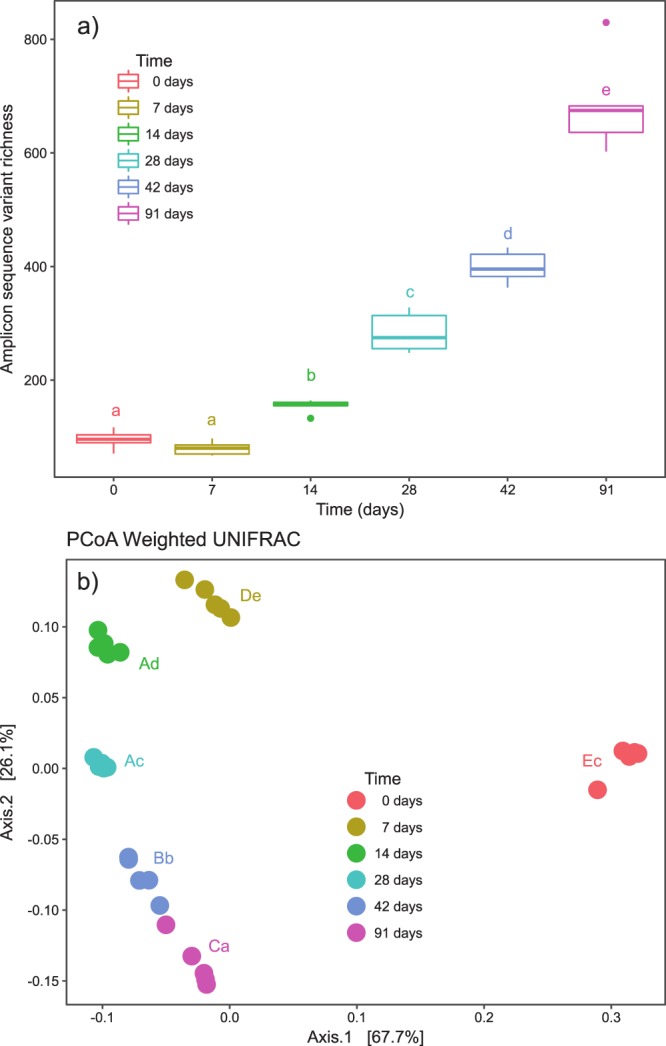
Changes in bacterial α-diversity and β-diversity during vermicomposting of the white grape marc *Vitis vinifera* v. Albariño. (**a**) α-diversity is described in terms of amplicon sequence variant (ASV) richness Letters indicate significant differences between time points (Tukey HSD test). (**b**) β-diversity is shown with principle coordinate analysis of weighted UniFrac distances. Capital and lowercase letters indicate significant differences between the time points in PCoA1 and PCoA2 scores respectively (Tukey HSD test, FDR corrected).

Bacterial communities in fresh white grape marc (day 0) had low α-diversity at both taxonomic (95.4 ± 7.7 ASVs) and phylogenetic levels (Faith PD 10.8 ± 1.2). Although α-diversity did not increase significantly between days 0 and 7, steady and significant increases were observed between days 7 and 91 in ASV richness, Chao1 richness, Shannon diversity (Fig. 3a–c), and Faith phylogenetic diversity (Supplementary Fig. 3). These increases in α-diversity are accompanied by also clear differences in phylogenetic and taxonomic β-diversity (Fig. 3d–f). Principle coordinate analysis demonstrated that fresh grape marc (day 0) bacterial composition was significantly different than that of vermicomposting grape marc (days 7–91). These trends were also evident using unweighted UniFrac, Bray-Curtis, and Jaccard distance matrices (Supplementary Fig. 3). Similar results were also found using close-reference OTUs (Supplementary Fig. 4).

### Core microbiome of white grape marc vermicomposting

**Figure 4:**
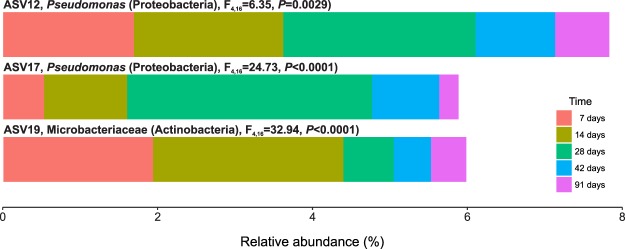
The core microbiome of vermicomposting the white grape marc *Vitis vinifera* v. Albariño. Initial substrate (day 0) was not considered for determination of the core microbiome. These three ASVs represent 8.56% of sequences from days 7 to 91, and are found in all samples (n = 25).

Although the bacterial community composition did not change drastically between days 7–91, only 3 ASVs were identified as part of the core microbiome in vermicomposting (Fig. 4). The initial substrate (day 0) was not included in the core microbiome since this sample was not subjected to the action of earthworms. The three ASVs identified were present in all samples (n = 25), and represent 8.56% of all sequences. Two of these ASVs belonged to the genus *Pseudomonas*, while the third ASV belonged to the family Microbacteriaceae.

### Functional diversity during vermicomposting of white grape marc

**Figure 5:**
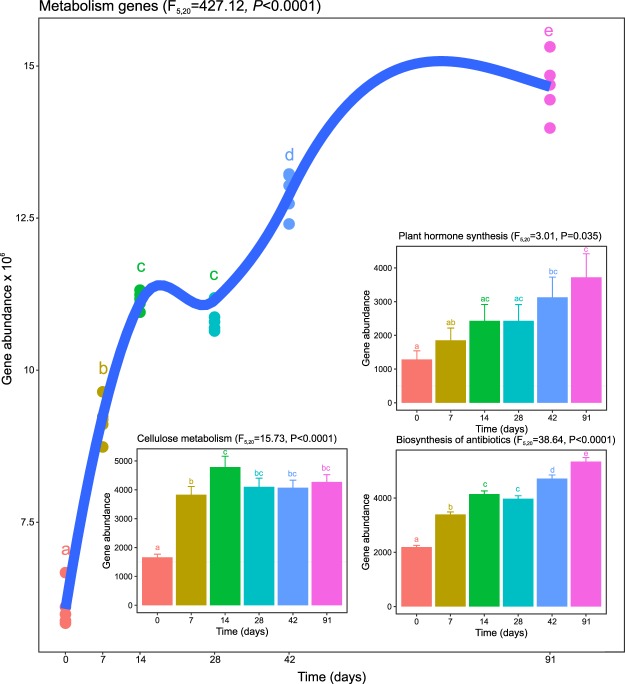
Changes in gene abundance of PICRUSt-predicted KEGG orthologies classified as “metabolism” in KEGG functional hierarchies during vermicomposting of the white grape marc *Vitis vinifera* v. Albariño. Individual values (n = 5) are plotted for each time point, and the curve was plotted using the “loess” smoothing method in ggplot2^52^. The insets show changes in gene abundance of all PICRUSt-predicted enzyme-level genes for synthesis of plant hormones and antibiotics, and cellulose metabolism. Values are presented as means ± standard error (n = 5). Above each plot, results from mixed-effects models are shown. Significance was determined using ANOVA, and the relevant F statistic (*F*_*5,20*_) and significance (*P*) are shown.

In addition to significant changes in bacterial community composition and diversity, metagenomic predictions using PICRUSt showed distinct microbiome functional profiles for different days of vermicomposting (Supplementary Fig. 4). More in detail, a significant increase (p < 0.001) in the number of genes classified as “metabolism” in KEGG functional hierarchies was observed over the course of the experiment (Fig. 5). In addition, the abundance of genes involved in plant hormone synthesis, antibiotic synthesis, and cellulose metabolism also increased during vermicomposting (Fig. 5, insets). No significant changes were detected in the abundance of genes related to polyphenol degradation such as polyphenol oxidases, peroxidases, laccases and lipoxygenases, despite the pronounced decrease in polyphenol content previously reported during vermicomposting of white grape marc^12^. However, the polyphenol content of white grape marc exponentially decreased with increasing α-diversity (Supplementary Fig. 5).

In addition, the identity and taxonomic affiliation of bacterial OTUs (phylum and genus level) contributing to gene counts in each category markedly differed over the course of the experiment (Supplementary Tables 1–3). For example, the majority of cellulose decomposition OTUs belonged to Proteobacteria (40%), Firmicutes (26%), Bacteroidetes (15%), and Actinobacteria (12%) at the beginning of the experiment (day 0). After earthworms began to process the grape marc, there was an increase in the contribution of Proteobacteria (Supplementary Table 1) and other bacterial phyla, which was accompanied by an increase in α-diversity (Fig. 2a, Table 1). Interestingly, Proteobacteria (52%) and Bacteroidetes (18%) were the main contributors to antibiotic synthesis gene abundance, and not Actinobacteria (12%) as might be expected (Supplementary Table 2). Similar results were observed for plant hormone synthesis (Supplementary Table 3).

## Discussion

Establishing sustainable agricultural practices in the wine industry necessitate better understanding of methods to recycle winery wastes such as grape marc. Vermicomposting of grape marc has previously been shown to successfully neutralize grape marc pH and reduce polyphenol content, resulting in a nutrient-rich organic fertilizer^12–14^. However, it is not well understood how the bacterial communities in vermicompost contribute to the stabilization of grape marc and affect the metabolic capacity of mature vermicompost. Vermicomposting was previously shown to alter bacterial communities in grape marc^18^, but, to the best of our knowledge, this is the first study to implement 16S rRNA sequencing to evaluate the bacterial succession during vermicomposting of grape marc.

There are relatively few studies describing bacterial succession during vermicomposting. It is well established that bacterial succession takes place during thermophilic composting^19–23^, but most vermicomposting studies have focused on mature vermicompost^24–27^. One previous study highlighted the changes in vermicompost bacterial communities during active and mature stages^22^, but did not evaluate bacterial succession throughout the active vermicomposting process. Recent work by our group with vermicomposting of the leguminous shrub Scotch broom captured multiple stages of the active vermicomposting process and demonstrated the clear bacterial succession that takes place during vermicomposting (Domínguez *et al*., submitted). The current study provides a detailed characterization of bacterial succession and functional diversity during vermicomposting of white grape marc.

Similar to previous findings with Scotch broom (Domínguez *et al*., submitted), the bacterial communities involved in vermicomposting of white grape marc change quickly and dramatically. Within the first seven days, the bacterial community composition changes significantly and continues to change for the duration of the experiment. This result highlights how quickly vermicomposting affects the bacterial communities from the initial substrate and emphasizes the importance of including early time points when studying bacterial succession during composting processes. Interestingly, although the phylum level composition is relatively similar from 7 days onward, the core microbiome of vermicomposting grape marc is quite small. Only 3 ASVs were consistently found in all samples from 7–91 days, making up approximately 8% of total sequences. This included two sequences from the genus *Pseudomonas*, which has been previously identified in compost and is thought to play a role in decomposition, denitrification, and plant disease suppression^20,28–30^. In addition to rapidly changing bacterial communities, bacterial diversity increases continuously from 7 to 91 days. These results are also similar to our previous findings in the vermicomposting of Scotch broom (Domínguez *et al*., submitted). In both cases, the diversity of the starting material was very low and had not previously been processed by a mammalian gut, unlike other frequently composted solid wastes such as manure or sewage. Vermicomposting of an already-diverse substrate may in fact reduce bacterial diversity due to the process of digestion in the earthworm gut. Since the dynamics of bacterial succession during vermicomposting are strongly influenced by starting substrate^31^, it is important to study the effect of a diverse range of vermicomposting substrates on bacterial community dynamics and functional capacities.

In addition to changes in bacterial community composition and increased diversity during vermicomposting of white grape marc, we observed a large increase in functional diversity (defined as PICRUSt-predicted KEGG orthologies). As indicated by the increase in gene abundance associated with the “metabolism” KEGG category, the metabolic capacity of the microbial community increased and diversified during vermicomposting (Fig. 5). In addition to a general increase in metabolism, there were increases in gene abundance related to specific metabolic processes. Genes related to cellulose metabolism increased in abundance within the first 7 days and remained high for the duration of the experiment. This is expected since cellulose degradation is an important component of the vermicomposting process. In a previous study, Domínguez, *et al*.^12^ found that cellulose and hemicellulose content decreased significantly during vermicomposting of grape marc. Our functional characterization provides support for the role of bacterial communities in cellulose and hemicellulose breakdown during vermicomposting.

Genes involved in plant hormone synthesis also increased significantly throughout the experiment. Previous work has found a higher concentration of plant hormones such as auxin, cytokinin, and gibberellin in vermicompost compared to compost^32^. Vermicompost has been shown to promote plant development, seed germination, flowering, and fruit production in a variety of plant species^33,34^, and the presence of plant hormones in vermicompost has been hypothesized to confer some of these benefits^35^. Our functional characterization indicates that the capacity for plant hormone synthesis increases during vermicomposting and supports the hypothesis that microbial-synthesized plant hormones are present in vermicompost, which may partially explain the positive effects of vermicompost on plant growth and development^33^. Moreover, vermicomposting of grape marc led to a significant reduction in polyphenol content and associated phytotoxicity, as previously described^12^. Unexpectedly, this was not associated with an increase in the abundance of genes involved in polyphenol degradation according to predictions by PICRUSt. One potential explanation is that earthworms can at least partially digest polyphenols^36^, which is supported by previous reports that polyphenol reduction peaks with maximum earthworm biomass^12,33^. Low polyphenol content in the final vermicompost product is crucial for its safe application as a soil amendment.

Genes involved in antibiotic synthesis also increased steadily throughout the vermicomposting of white grape marc. In addition to enhancing plant growth and development, vermicompost has been shown to mitigate or suppress plant diseases^33,35^. Although the precise mechanism behind this disease suppression is unknown, antibiotic production by soil microbes has been hypothesized to provide this benefit^37^. The increase in genes relating to antibiotic synthesis during vermicomposting supports this hypothesis and warrants additional research on the relationship between antibiotic content in vermicompost and plant disease resistance.

As expected, most of the OTUs involved in cellulose metabolism, plant hormone synthesis, and antibiotic synthesis belonged to *Proteobacteria* and *Bacteroidetes*. However, we found that the genera performing these functions varied over the course of the experiment, indicating that there was succession in the bacteria responsible for these functional roles.

Although the beneficial effects of vermicompost on plants have been well described, the mechanisms behind these effects are less well understood. Furthermore, there is evidence that the vermicompost application can have variable effects on plant growth, or even in some cases have harmful effects^33^. Variation in the metabolic functions of vermicompost microbiomes may contribute to these variable effects. The vermicompost microbiome has been shown to vary with different vermicomposting methods, earthworm species, and initial substrates^25,31,38^. Therefore, it is not known whether all vermicompost bacterial communities will have the same functional capacity and thus confer the same benefits to plants. Understanding the relationship between the initial substrate, bacterial communities, and their metabolic capacity may be important for understanding the benefits of vermicompost. Therefore, future work should evaluate the bacterial communities and succession involved in vermicomposting of a diverse range of substrates to further our understanding of the metabolic capacity of different vermicomposts.

## Conclusions

This study provides the first characterization of bacterial succession during vermicomposting of white grape marc using 16S rRNA high-throughput sequencing. Our results indicate that vermicomposting quickly and significantly alters bacterial community composition and increases bacterial diversity. These changes are accompanied by increases in the metabolic capacity of the bacterial community, and increases in specific metabolic processes including cellulose metabolism, antibiotic synthesis, and plant hormone synthesis. These results provide novel evidence for microbial-derived benefits for plants grown in soil amended with vermicompost and supports previous findings that vermicomposting of grape marc may be a sustainable and beneficial practice for the winery industry.

## Methods

### Grape marc

White grape marc (*Vitis vinifera* v. Albariño) was provided by the Mar de Frades winery located in Pontevedra (Galicia, NW Spain) and stored at 4 °C until use. The grape marc was turned and moistened with water during two days prior to the trial in order to achieve a suitable level of moisture (85%) for the earthworms.

### Vermicomposting set-up and sampling design

Vermicomposting was performed in a rectangular metal pilot-scale vermireactor (4 m long × 1.5 m wide × 1 m high). The vermireactor was housed in a greenhouse with no temperature control. A 12 cm layer of vermicompost was used as a bed for the earthworms (*Eisenia andrei*) before adding the grape marc. The initial earthworm population density in the vermireactor was 297 ± 20 individuals m^−2^, including 19 ± 3 mature earthworms m^−2^, 215 ± 37 immatures m^−2^ and 63 ± 18 cocoons m^−2^, with a mean biomass of 58.4 ± 15 g m^−2^. Fresh grape marc (158 kg fresh weight) was added to the bed in a 12 cm layer. A plastic mesh (5 mm mesh size) was used to divide the vermicompost bedding from the fresh grape, allowing for earthworm migration and facilitating sampling of the grape marc, but preventing the mixing of processed grape marc and vermicompost bedding. The density and biomass of the earthworm population were determined every 14 days during the trial (91 days) by collecting 10 samples (five from above and five from below the plastic mesh) of the material in the vermireactor. The samples were collected with a core sampler (7.5 cm diameter and 12 cm height).

For sampling of microbial activity and composition, the grape marc layer was divided into 5 sections, and two samples (10 g) were taken at random from each section at the beginning of the experiment and after 7, 14, 28, 42 and 91 days of vermicomposting. The two samples from each section were combined and stored in plastic bags at −80 °C until analysis.

### Microbial activity

Microbial activity was determined by measuring the oxygen consumption using a WTW OxiTop® Control System (Weilheim, Germany) according to ISO 16072^39^.

### DNA sequencing and analysis

DNA was extracted from 0.25 g (fresh weight) of grape marc using the MO-BIO PowerSoil® kit following the manufacturer’s protocols. DNA quality and quantity were determined using BioTek’s Take3™ Multi-Volume Plate. All laboratory procedures were performed under a laminar flow hood to prevent contamination of the samples with microorganisms from the surrounding environment. We sequenced a fragment of the 16S rRNA gene V4 region using a dual-index sequencing strategy^40^ on the Illumina MiSeq platform at the Genomics Core Facility of the Universitat Pompeu Fabra (Barcelona, Spain). Five samples from each sampling time (0, 7, 14, 28, 42 and 91 days) were sequenced for a total of 30 samples.

The DADA2 pipeline (version 1.8) was used to infer the amplicon sequence variants (ASVs) present in each sample^41^. Standard filtering parameters were used, with forward reads truncated at 230 nt and reverse reads at 160 nt, and a maximum of 2 expected errors per read. Default settings were used for ASV inference and chimera detection. Taxonomy was assigned against the Silva v132 database using the *assignTaxonomy* function in dada2 with a minimum bootstrap confidence of 80^42,43^. An average of 50,023 ± 12,560 sequences per sample passed all quality filters, for a total of 1,500,694 sequences across all samples. These sequences were assigned to 3,725 ASVs without singletons and doubletons. All samples were rarefied to 20,267 sequences in order to normalize the number of sequences. Rarefaction curves indicated that this sampling depth was sufficient for our samples (Supplementary Fig. 1). After rarefaction, sequences were assigned to a total of 1,967 ASVs without singletons and doubletons.

Functional diversity of the bacterial communities was predicted using the Phylogenetic Investigation of Communities by Reconstruction of Unobserved States software package (PICRUSt)^44^. Closed reference operational taxonomic units (OTUs) were identified using the 13_5 version of Greengenes database at 97% identity. The resulting OTU table was then normalized for copy number variation and used to predict functions for metagenomes according to the PICRUSt workflow. The weighted nearest sequenced taxon index (NSTI) for our samples was 0.10 ± 0.07 (mean ± s.d.), similar to that observed in soil samples, indicating that PICRUSt is expected to produce reliable results^44^. Predicted metagenomes were collapsed using the Kyoto Encyclopedia of Genes and Genomes (KEGG) Pathway metadata. Metagenome functional contributions were partitioned according to function, OTU, and sample to evaluate how the OTUs responsible for specific functional roles change during vermicomposting.

### Statistical analysis

Taxonomic α-diversity was described as the number of observed ASVs, and diversity and richness were estimated with Shannon and Chao1 indexes, respectively. An approximately maximum-likelihood phylogenetic tree was inferred using FastTree 2.1^45^, and phylogenetic diversity was calculated as Faith’s phylogenetic diversity^46^. Taxonomic β-diversity at the ASV level was described by performing principal coordinate analysis (PCoA) using Bray-Curtis and Jaccard distance matrices. Likewise, phylogenetic β-diversity was estimated by PCoA using weighted and unweighted unifrac matrix distances^47^ in the ‘phyloseq’ R package^48^. Mixed models were implemented with the ‘nlme’ R package^49^ to analyze the effect of time on α- and β-diversity (PCoA scores) of bacterial communities from the grape marc, with time as the fixed factor. Repeated measures were accounted for by considering the effect of time nested in each sample as a random factor. The normality of residuals and homogeneity of variance across groups was checked for each variable. Post-hoc comparisons were performed with Tukey’s test, and multiple test corrections were performed with Benjamini–Hochberg FDR implemented in the ‘multcomp’ R package^50^. The same model was used to analyze the effect of time on the relative abundances of bacterial phyla and classes.

Additionally, mean relative abundance of gene contents from PICRUSt analysis and collapsed using KEGG pathway metadata were analyzed with mixed models and post-hoc test as described above. As an additional measure of β-diversity we carried out PCoA analysis with Bray-Curtis and Jaccard distance matrices using data from functional (gene abundances) and taxonomic (normalized closed referenced OTUs) composition of those metagenomes.

All analyses were performed in R version 3.5^51^. Figures were created using the R package ggplot2^52^.

## Supplementary information

Supplementary Figures 1-5

Supplementary Tables 1-3

## Data Availability

The sequence data generated in the current study are available in the GenBank SRA database under accession SRP171648.
