# Supplementary material for: Bacterial succession and functional diversity during vermicomposting of the white grape marc Vitis vinifera v. Albariño
Source: Sci Rep. 2019 May 16;9:7472. doi: 10.1038/s41598-019-43907-y (PMC6522490; doi:10.1038/s41598-019-43907-y)
Supplement: Supplementary file 1 — Supplementary Figures 1-5 [file 41598_2019_43907_MOESM1_ESM.pdf]

Bacterial succession and functional diversity during vermicomposting of the white grape marc *Vitis vinifera* v. Albariño

Supplementary Figures

Allison R. Kolbe, Manuel Aira, María Gómez-Brandón, Marcos Pérez-Losada, and Jorge Domínguez

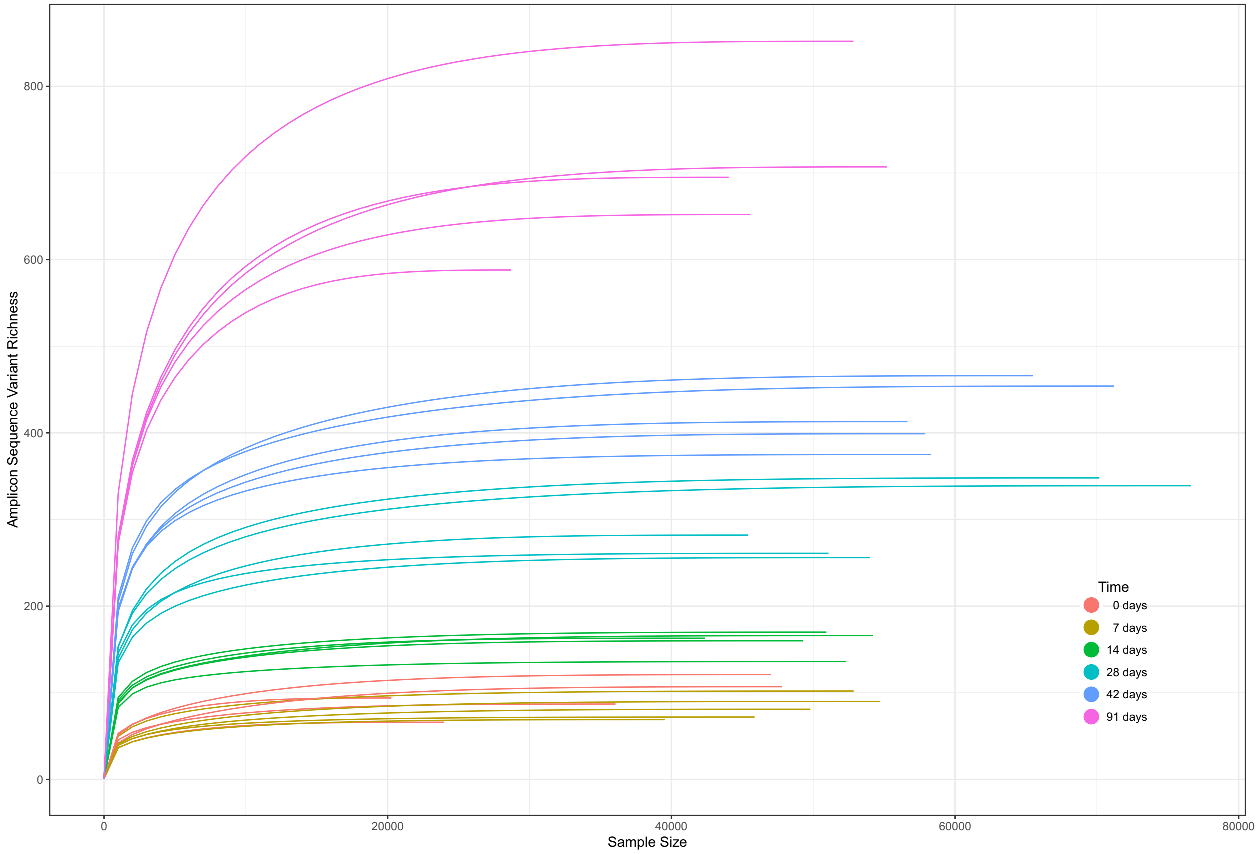

Figure S1: Rarefaction curves indicating the number of amplicon sequence variants (ASVs) found in each sample during vermicomposting of the white grape marc *Vitis vinifera* v. Albariño. These curves indicate that subsampling depth (20,267) was optimal for all samples.

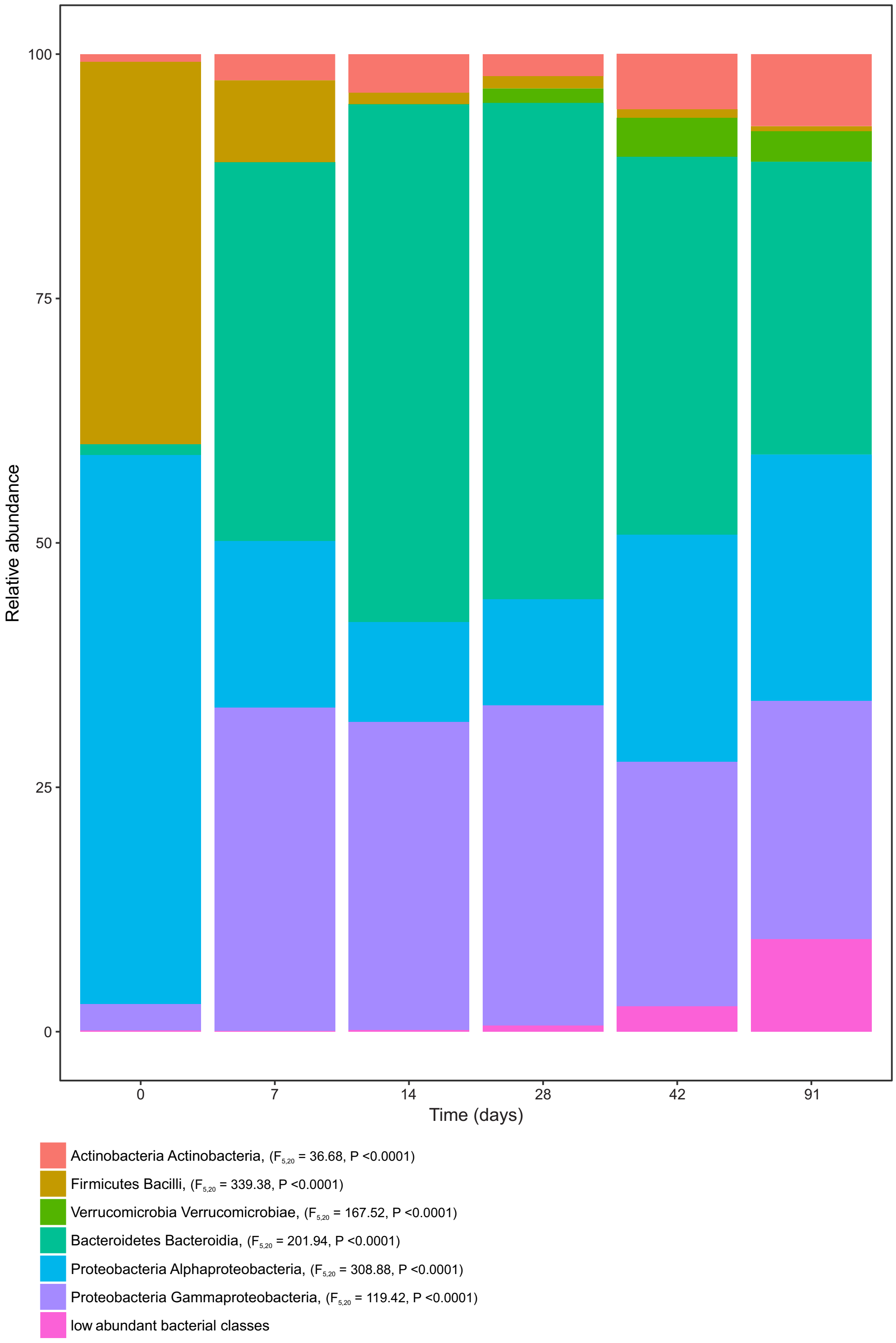

Figure S2: Bacterial community composition (class level) during vermicomposting of the white grape marc *Vitis vinifera* v. Albariño, presented as mean relative abundance at each time point. Low abundance bacterial classes (<1%) were grouped. Results from mixed-effects models analyses are shown, and significance was calculated using ANOVA. For each bacterial class, the relevant *F*-statistic ($F_{5,20}$) and p-value are shown.

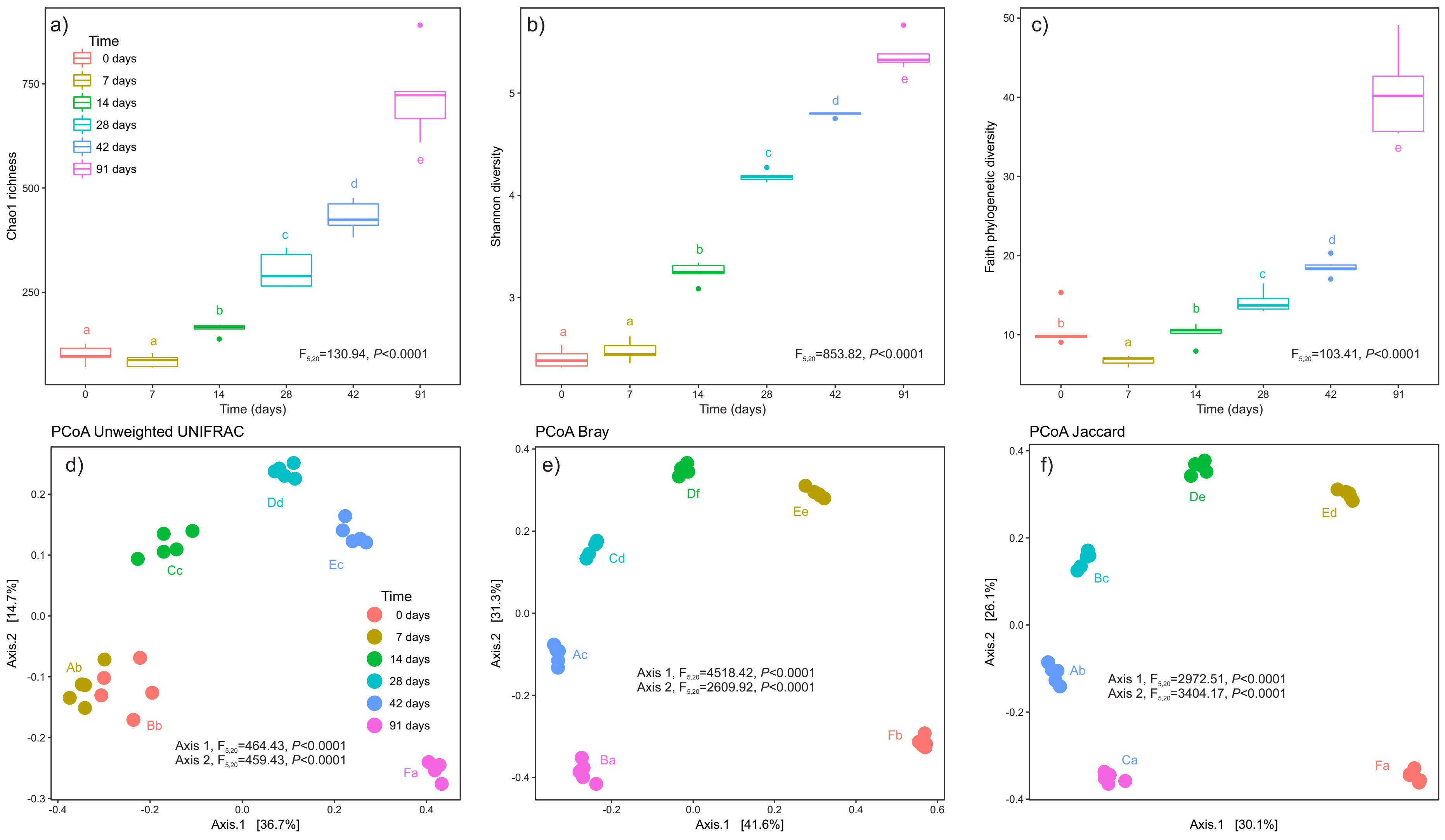

Figure S3: Additional estimates of bacterial α-diversity and β-diversity during vermicomposting of the white grape marc *Vitis vinifera* v. Albariño. α-diversity increased over time regardless of estimation method, including Chao1 richness (a), Shannon diversity (b), and Faith phylogenetic diversity (c). Letters in (a)-(c) denote significant differences between the different stages of the vermicomposting process (Tukey HSD test). Principle coordinate analysis with unweighted UniFrac (d), Bray-Curtis (e), and Jaccard (f) showed significant differences between stages of vermicomposting. Different capital and lowercase letters indicate significant differences between the time points in PCoA1 and PCoA2 scores, respectively.

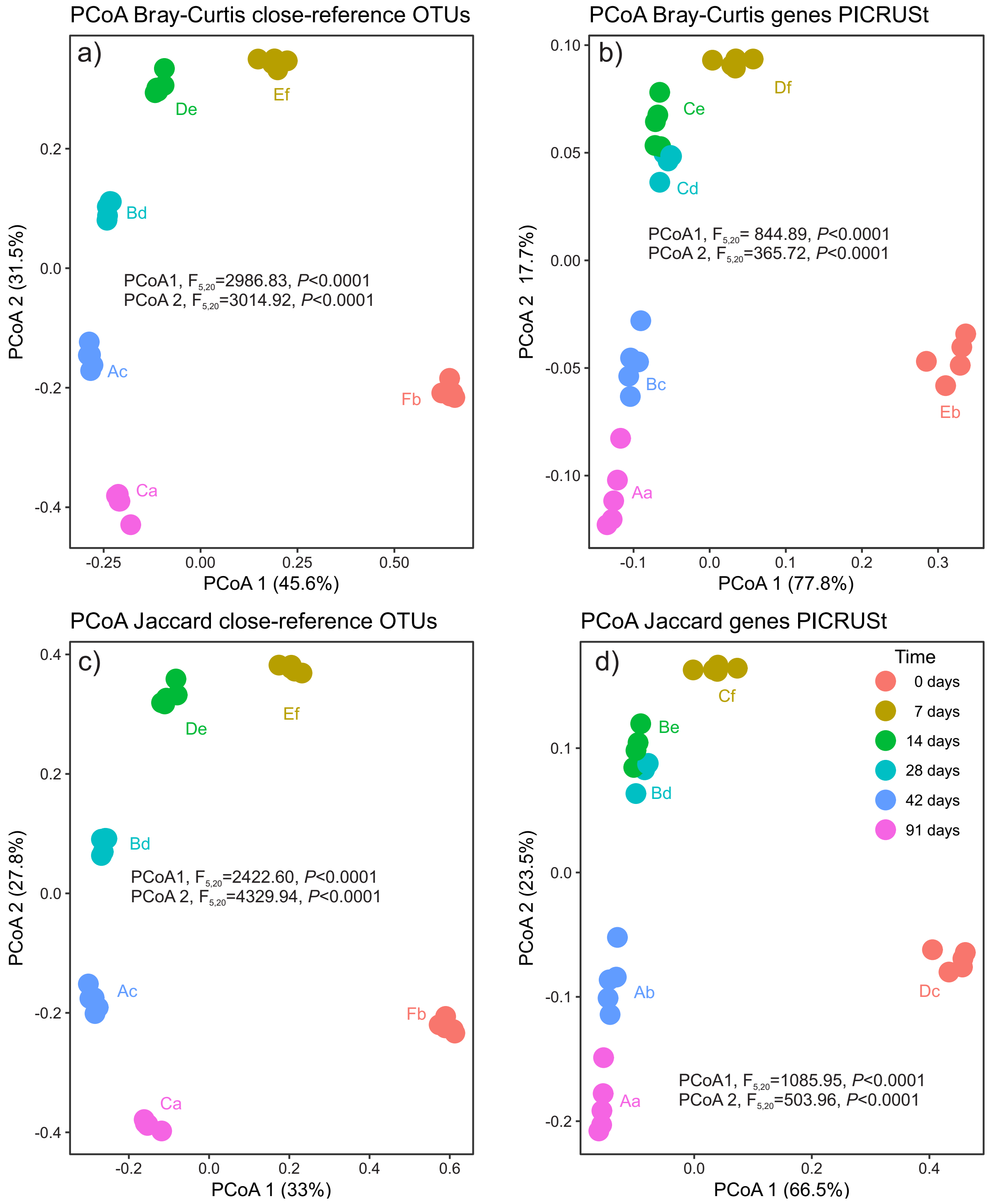

Figure S4: Additional estimates of β-diversity during vermicomposting of the white grape marc *Vitis vinifera* v. Albariño using close-reference OTUs and functional profiles. Bray-Curtis dissimilarity matrices were used in principle coordinate analysis with close-reference OTUs (a) and functional profiles generated by PICRUSt (b). Similarly, Jaccard dissimilarity matrices were used in principle coordinate analysis with close-reference OTUs (c) and functional profiles generated with PICRUSt (d). Capital and lowercase letters indicate significant differences between the time points in PCoA1 and PCoA2 scores, respectively.

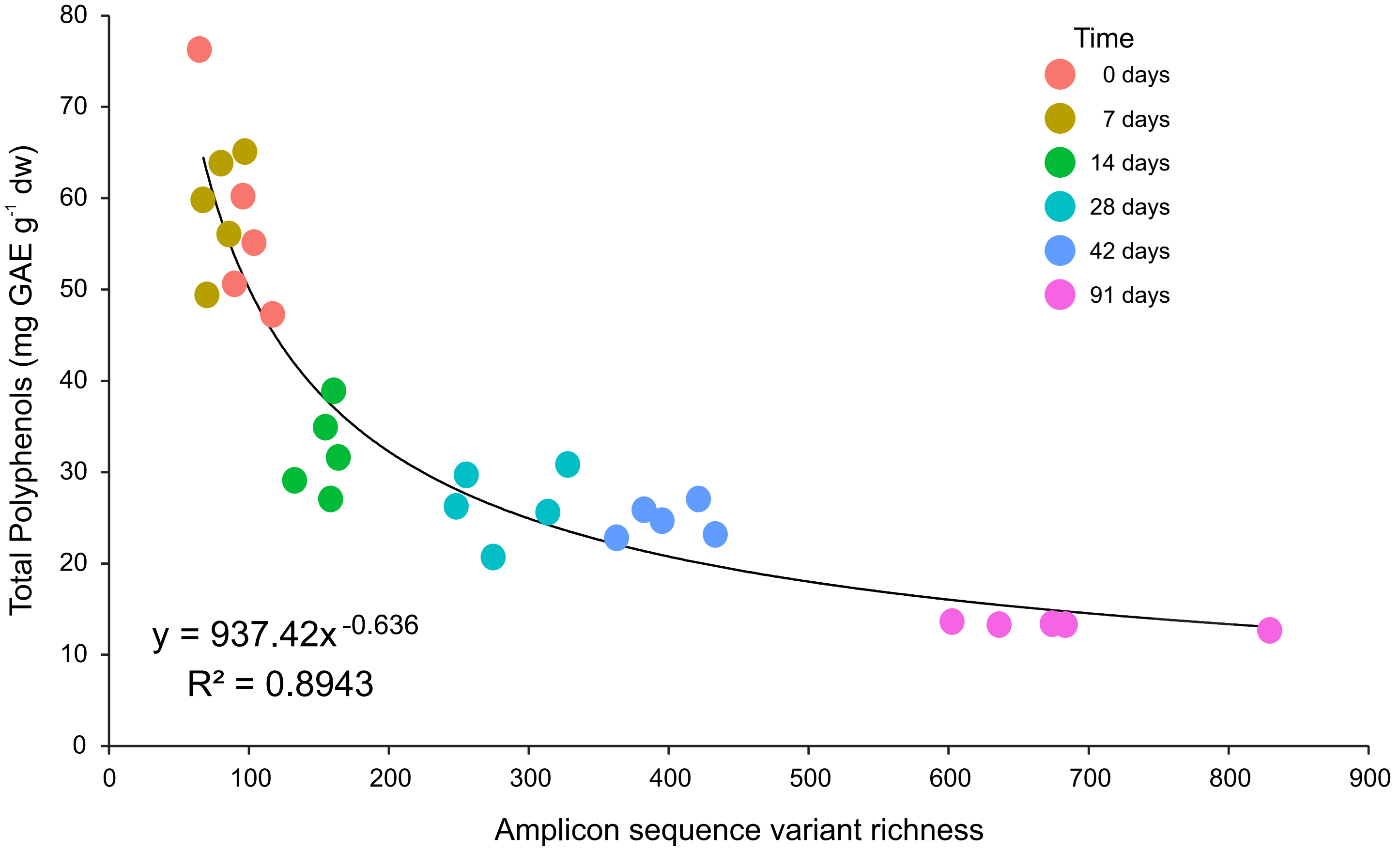

Figure S5: Regression analysis between the total polyphenol content and amplicon sequence variant (ASV) richness during vermicomposting of white grape marc *Vitis vinifera* v. Albariño. Polyphenol content data was previously presented in Domínguez et al., 2014 (Waste Management & Research, Vol. 32(12) 1235–1240).
